# Neuronal mechanisms and circuits underlying repetitive behaviors in mouse models of autism spectrum disorder

**DOI:** 10.1186/s12993-016-0087-y

**Published:** 2016-01-20

**Authors:** Hyopil Kim, Chae-Seok Lim, Bong-Kiun Kaang

**Affiliations:** Department of Biological Sciences, College of Natural Sciences, Seoul National University, 1 Gwanangno, Gwanak-gu, Seoul, 08826 South Korea

**Keywords:** Autism spectrum disorder, ASD mouse models, Repetitive behaviors, Cortico-basal ganglia-thalamic circuits

## Abstract

Autism spectrum disorder (ASD) refers to a broad spectrum of neurodevelopmental disorders characterized by three central behavioral symptoms: impaired social interaction, impaired social communication, and restricted and repetitive behaviors. However, the symptoms are heterogeneous among patients and a number of ASD mouse models have been generated containing mutations that mimic the mutations found in human patients with ASD. Each mouse model was found to display a unique set of repetitive behaviors. In this review, we summarize the repetitive behaviors of the ASD mouse models and variations found in their neural mechanisms including molecular and electrophysiological features. We also propose potential neuronal mechanisms underlying these repetitive behaviors, focusing on the role of the cortico-basal ganglia-thalamic circuits and brain regions associated with both social and repetitive behaviors. Further understanding of molecular and circuitry mechanisms of the repetitive behaviors associated with ASD is necessary to aid the development of effective treatments for these disorders.

## Background

Autism spectrum disorder (ASD) is a neurodevelopmental disorder, and according to the 5th edition of the Diagnostic and Statistical Manual of Mental Disorders (DSM-5), it is characterized by “an impairment in social communication and interaction, and restricted and repetitive patterns of behaviors, interests or activities.” A subset of patients with ASD tend to also display hyperactivity, anxiety, hypotonia, epilepsy, sensory abnormalities, sleep disorders, intellectual disabilities, gastrointestinal disorders, microencephaly or megalencephaly [1–4]. The symptom severity is also heterogeneous, which is why the term ASD is used to encompass all the different severities and variations of symptoms in these disorders.

ASD patients show a range of repetitive behaviors, including stereotypies, rituals, compulsions, obsessions and self-injurious. The repetitive behaviors of each patient are not always convergent. Moreover, repetitive behaviors appear in many other neurodevelopmental and neuropsychiatric disorders [5]. Therefore, it is hard to distinguish repetitive behaviors of ASD patients from those of other neuropsychiatric disorders, like obsessive–compulsive disorder (OCD) [6]. However, as the diagnostic criteria implying, the repetitive behaviors of ASD patients may be related with social deficits. Supporting this, some treatments that rescued social deficits could restore repetitive behaviors, too. For example, oxytocin, a neuropeptide, promoted social interaction while reducing repetitive behaviors of ASD patients [7, 8].

Today, ASD draws global attention as the number of people diagnosed has rapidly increased. According to epidemiological studies, the current prevalence of autism is estimated to be 10–30 per 10,000 people, with ASD occurring in an estimated 69.5 per 10,000 people [9]. Specifically, a study reported that the prevalence of ASD in Asia was as high as 264 per 10,000 people (about one out of 38 people) [10]. Among psychiatric diseases, this is relatively high incidence rate, which is why many researchers and clinicians are trying to understand the pathophysiology of ASD.

Since the direct analysis of human patients is difficult for in vivo and biochemical experiments, ASDs are currently studied using animal models. Among the available model organisms, mouse models are most commonly used in ASD researches, because of the ease of genetic manipulation, high accessibility, and relatively high similarity to humans. A number of transgenic ASD mouse models have been generated based on genomic studies of patients with ASD, the findings from which indicate that genomic components underlie the various ASD phenotypes [11–15]. They display various types of repetitive behaviors [11–13, 16, 17], and mice with a higher level of stereotyped behaviors also display more restricted behavioral patterns and diminished flexibility in learning, reversal learning, and extinction tasks [18, 19].

Although many scientists have explored the mechanisms of repetitive behaviors, the specific genetic and molecular factors underlying these repetitive behaviors and how they interact with each other in distinct brain regions are yet to be elucidated. Each characteristic repetitive behavior seems to stem from different molecular mechanisms and distinct neuronal pathways. In this review, we summarize the types of repetitive behaviors and neurophysiological properties of them associated with different ASD mouse models and compare the functions of affected genes for repetitive behaviors within each brain region. This review should aid in the understanding of the molecular and circuitry mechanisms underlying repetitive behaviors in ASD and contribute to the development of effective and targeted therapies for the repetitive behaviors associated with ASD.

## Repetitive behaviors of ASD mouse models

A detailed understanding of the neural mechanisms underlying ASD is hampered by its complexity and heterogeneity. ASD is associated with abnormalities in various brain regions such as the neocortex, hippocampus, amygdala, and basal ganglia, which mediate social interaction, communication and repetitive behaviors.

Focusing on repetitive behaviors [20], despite the lack of specific criterion to define each repetitive behavioral category, according to Turner, repetitive behavior should be repeated at a relatively high rate, pursued in an invariant way, and considered inappropriate and abnormal in its manifestation and display. For example, just opening and closing a door to enter a room is not regarded as a repetitive behavior, even though the behavior is normally repeated every day. However, if someone open and close a door continuously without a specific purpose, then it could be classified as a repetitive behavior. Thus, a repetitive behavior can be heterogeneous depending on its characteristics and circumstances. Similarly, ASD mouse models also show several types of stereotyped repetitive behaviors including self-grooming, jumping, circling and marble burying. Example movies of each behavior are available in the previously published papers [12, 21–23]. A specific repetitive behavior of an ASD mouse model would depend on the characteristic abnormal physiological features resulting from an altered gene expression or a modification of a gene product. The detailed characteristics of ASD mouse models are summarized in (Table 1).

**Table 1:** Repetitive behaviors and neuronal phenotypes in ASD mouse models

Type of repetitive behaviors	Model mouse	Biological functions of the target protein	Other behavioral characteristics	Implicated brain regions and neural phenotypes	Rescue of repetitive behaviors
Self-grooming	BTBR T + tf/J (inbred strain) [16]		Hyperactive Impaired cognitive flexibility [83, 84]	Decreased sIPSC and increased sEPSC frequency in the hippocampus [27] No corpus callosum [24]	Repetitive self-grooming and E/I balance with GABA-R agonists such as clonazepam [34] Repetitive self-grooming with mGluR5 antagonism [32, 33] Repetitive self-grooming with a NMDA-R agonist, d-cycloserine [85]
	*Scn1a* ^+/−^ [13]	A voltage-gated sodium channel NaV1.1 as a primary sodium channel specifically in GABAergic neurons	Hyperactive [13]	Decreased sIPSC and increased sEPSC frequency in the hippocampus and prefrontal cortex [13]	
	*Cntnap2* ^−/−^ [28]	A kind of neurexin family, implicated in neuron-glia interactions and K+ cahnnel clustering [28]	Hyperactive [28]	Decreased GABAergic neurons in the corpus callosum, somatosensory cortex, and striatum [28]	Repetitive self-grooming with a D2R antagonist, risperidone [28]
	*NRXN1α* ^−/−^ [86]	A presynaptic protein that binds to postsynaptic protein, neuroligin, forming and strengthening synapses	Hypoactive [87]	Decreased mEPSC frequency in the hippocampus [86]	
	Expression of dominant negative mutation of neurexin 1β [88]			Decreased mEPSC and mIPSC frequency in the somatosensory cortex [88]	
	S*hank3b* ^−/−^ [34]	A scaffolding and postsynaptic density (PSD) protein found at glutamatergic synapses, forming complexes with PSD95, SAPAP, Homer, and GKAP Implicated in 22q13 deletion syndrome (Phelan–McDermid syndrome),	Self-injurious grooming Decreased rearing [34]	Decreased cortico-striatal pop-spike amplitude [34]	
	*En2* ^−/−^ [89]	A transcription factor which is important for development, including neuronal differentiation of the mid/hind brain [90]	Hyperactive (only p21–27) [89]	Decreased GABAergic neurons in the hippocampus and cerebral cortex [90]	
	*Ephrin*-*A* ^−/−^ *Ephrin*-*A3* ^−/−^ [91]	A ligand of cell-surface ephrin receptor implicated in development and synaptic plasticity of neurons [92, 93]	Self-injurious grooming Hypoactive Decreased rearing [91]	`	
	*Shank1* ^−/−^ [94]	A scaffolding and PSD protein found at glutamatergic synapses, forming complexes with PSD95, SAPAP, Homer, and GKAP	Hypoactive Impaired rotarod performance [94]	Smaller dendrites and thinner PSDs in hippocampal neurons Decreased mEPSC in the hippocampus Decreased input–output (I/O) curve in the hippocampus [94]	
	*Eif4ebp2* ^−/−^ [95]	Initiation of translation by leading eukaryotic mRNA to ribosomes		Increased mEPSC amplitude and frequency Increased mIPSC amplitude Increased net E/I ratio in the hippocampus [95]	
	*Viaat* (vesicular inhibitory amino acid transporter)-*Mecp2* ^−/y^ [96]	Suppression or activation of target genes and implicated in Rett syndrome	Hypoactive [96]	Decreased mIPSC amplitude in cortical slices Impaired LTP in the hippocampus Mecp2 depletion in inhibitory neurons [96]	
Jumping	C58/J [32]		Hyperactive Hypersensitivity to amphetamine [17, 32]		Repetitive jumping with a mGluR5 negative allosteric modulator, GRN-529 [25]
	*Shank2* ^−/−^ [12]	A scaffolding and PSD protein found at glutamatergic synapses, forming complexes with PSD95, SAPAP, Homer, and GKAP	Hyperactive Decreased digging [12]	Decreased NMDA/AMPA ratio Impaired LTP and LTD in the hippocampus [12]	
	NL2 overexpression [79]	An adhesion molecule binding with presynaptic neurexins, regulating excitatory and inhibitory synaptic functions		Enlarged synaptic contact size Increased mIPSC frequency in PFC Decreased E/I balance in PFC [79]	
Circling	*Scn1a* ^+/−^ [13]		Hyperactive [13]	Decreased sIPSC and increased sEPSC frequency in the hippocampus and prefrontal cortex [13]	
	*Gabrb3* ^−/−^ [97]	A subunit of GABA-R, a chloride channel, implicated in ASD and seizure	Decreased rearing [97]	Hypoplasia of the cerebellar [97]	
Decreased marble burying	*Shank1* ^−/−^ [39]		Decreased rearing [39]		
	*Ephrin*-*A* ^−/−^ *Ephrin*-*A3* ^−/−^ [91]	A ligand of cell-surface ephrin receptor implicated in development and synaptic plasticity of neurons [92, 93]	Self-injurious grooming Hypoactive Decreased rearing [91]	`	
	C58/J) [32]		Hyperactive Hypersensitivity to amphetamine [17, 32]		
	Glutamate receptor, ionotropic, N-methyl D-aspartate 1 (*Grin1*) knock-down [11]	An NMDA-R subunit implicated in synaptic plasticity	Hyperactive Decreased anxiety [72]		
Increased marble burying	BTBR [16]		Hyperactive Impaired cognitive flexibility [83, 84]	Decreased sIPSC and increased sEPSC frequency in the hippocampus [27] No corpus callosum [24]	
	*Eif4ebp2* ^−/−^ [95]	Initiation of translation by leading eukaryotic mRNA to ribosomes		Increased mEPSC amplitude and frequency Increased mIPSC amplitude Increased net E/I ratio in the hippocampus [95]	
	*FMR1* ^−/−^ [98]	Regulation of hundreds of mRNAs in the synapses Depletion of FMR1 leads to fragile X syndrome	Hyperactive [99]	Hyper-excitability caused by decreased activities of fast-spiking (FS) inhibitory neurons in the somatosensory, barrel cortex. [100]	
Rearing	C58/J [32]		Hyperactive Hypersensitivity to amphetamine [11, 24]		
Head poking	*Shank3* ^−/−^ [101]		Hypoactive [101]	Increased spine length in the hippocampus Decreased mIPSC frequency and Impaired LTP in the hippocampus [101]	
Forelimb movements	Expression of truncated Mecp2 [102]	Suppression or activation of target genes and implicated in Rett syndrome	Hypoactive Motor deficits [102]		
	*Viaat*-*Mecp2* ^−/y^ [96]			Decreased mIPSC amplitude in cortical slices Impaired LTP in the hippocampus Mecp2 depletion in inhibitory neurons [96]	
Hanging	Expression of SERT Ala56 [103]	Returns serotonin excreted into the synaptic cleft to the presynaptic boutons		Decreased firing in dorsal raphe neurons Hyperserotonemia [103]	

Although the ASD mouse models show a variety of alterations in molecular level, including metabotropic glutamate receptor 5 (mGluR5), *N*-methyl-D-aspartate glutamate receptor (NMDA-R) and γ-Aminobutyric acid receptor (GABA-R), a specific molecular pathway seems to be related with a specific repetitive behavior. Dopamine also could affect a repetitive behavior by modulating dopamine receptors in the striatum (Table 1).

### Self-grooming

Mice normally scratch and brush their hair with their forelimbs for a few seconds to minutes. However, when the self-grooming behavior is repeated at a higher rate and a longer duration, it can be considered as a repetitive behavior. For example, BTBR T + tf/J (BTBR) mice, an inbred strain without corpus callosum, are used as a model of idiopathic autism with an increased rate of repetitive self-grooming [24]. This excessive self-grooming was rescued by inhibiting mGluR5 activity [25, 26]. Many transgenic mouse models of ASD also display repetitive and excessive self-grooming and the functions of many neurotransmitters are implicated in this behavior (Table 1). For example, GABA-R agonists reduce repetitive self-grooming in BTBR mice without sedation [13, 27]. Abnormal self-grooming also occurs in mice with contactin-associated protein-like 2 (*Cntnap2*)^−/−^ mice, which was also rescued by treatment with risperidone, an antagonist to the dopamine D2 receptor (D2R) [28]. While we do not yet know the exact mechanisms underlying repetitive self-grooming, we do know that it is associated with multiple brain regions including the cortex, hypothalamus, striatum, cerebellum and amygdala [29–31].

### Jumping

Another repetitive behavior observed in rodent ASD models is jumping. Most mice including C57BL/6 mice, a normal control mouse frequently used in behavioral researches sometimes jump using their hind limbs. In contrast with C57BL/6 mice, the C58/J mice are characterized by excessive jumping [32]. Interestingly, while SH3 and multiple ankyrin repeat domains 3b (*Shank3b)* ^−/−^ mice display repetitive grooming, the *Shank2* ^−/−^ mice exhibit repetitive jumping and scrabbling behaviors (Table 1), despite the functional similarities between Shank3 and Shank2 proteins. Both proteins are enriched in excitatory synapses as scaffolding proteins, and their deficiency reduces NMDA-R activity [12, 33]. However, only Shank3, but not Shank2, is highly expressed in the cerebellum and striatum, implicating different functions of the two proteins in distinct brain regions [34]. Interestingly, inhibition of mGluR5 signaling through GRN-529, a negative allosteric modulator of mGluR5, reduces the jumping behavior of C58/J mice [24]. NMDA-R hyperfunction may be responsible for triggering the jumping observed in C58/J mice, because inhibition of mGluR5 signaling can alter NMDA-R activity and both receptors are tightly connected in the postsynaptic density (PSD).

### Circling

Some ASD mice repeatedly rotate in fixed locations, in a circular pattern. Dopaminergic pathways from the striatum to the substantia nigra (SN) are implicated in mediating the circling behaviors, and an imbalance in striatal dopamine activity is thought to be a cause of the abnormal circling behaviors [35–37]. The direction of rotation seems to be contralateral to the brain region with high striatal dopaminergic activity. For example, when the dopamine D1 receptor (D1R) agonist, A68930, was injected in the striatum of one hemisphere of Bronx Waltzer mice, which show repetitive circling behaviors, their contralateral rotation increased while their ipsilateral rotation decreased [37].

Since alterations in GABA levels can also impair the homeostatic activity levels in the brain, GABA may also affect circling behaviors. For example, muscimol, a GABA-R agonist, injected into the substantia nigra pars reticulata (SNr) induced a repetitive circling behavior [36].

### Marble burying

Marble burying is the behavior of burying marbles scattered on the bedding into the bedding. It is a little controversial to categorize marble burying as a repetitive behavior, since the behavior is associated with anxiety to a novel context and exploration. Consistent with this, some ASD mouse models show increased marble burying behavior, while others demonstrate a decrease (Table 1). Interestingly, some mice with increased marble burying also exhibit increased locomotor activity, while other mice with decreased marble burying exhibit decreased locomotor activity [38, 39]. This suggests the possibility of an overlap between the neuronal circuits involved with digging behavior and locomotor activity. In addition, several antidepressants/anxiolytics such as fluvoxamine, bupropion, and diazepam reduced both marble burying and digging [40]. Furthermore, minocycline, which shows an anxiolytic effect on Fragile X mental retardation 1 (*Fmr1*) KO mice, also reduced the elevated marble burying in these mice. Thus, the emotional states and brain circuitry involved with anxiety or depression may also affect marble burying behaviors.

### Hyperactivity

Hyperactivity itself is not generally considered as a repetitive behavior, but the phenotype is often accompanied by repetitive body movements. Moreover, several ASD mouse models exhibit hyperactivity (Table 1). Supporting the relationship between repetitive behaviors and hyperactivity, local field potential recordings in several interconnected brain regions of norepinephrine-deficient mice, which display hyperactivity and repetitive grooming, indicated an impaired coherence across the cortico-striatal circuits [41].

Other ASD mouse models in addition to the aforementioned mouse models, show additional repetitive behaviors, including rearing, head poking, forelimb movements and hanging (Table 1).

## General neural circuits of repetitive behaviors

**Fig. 1:**
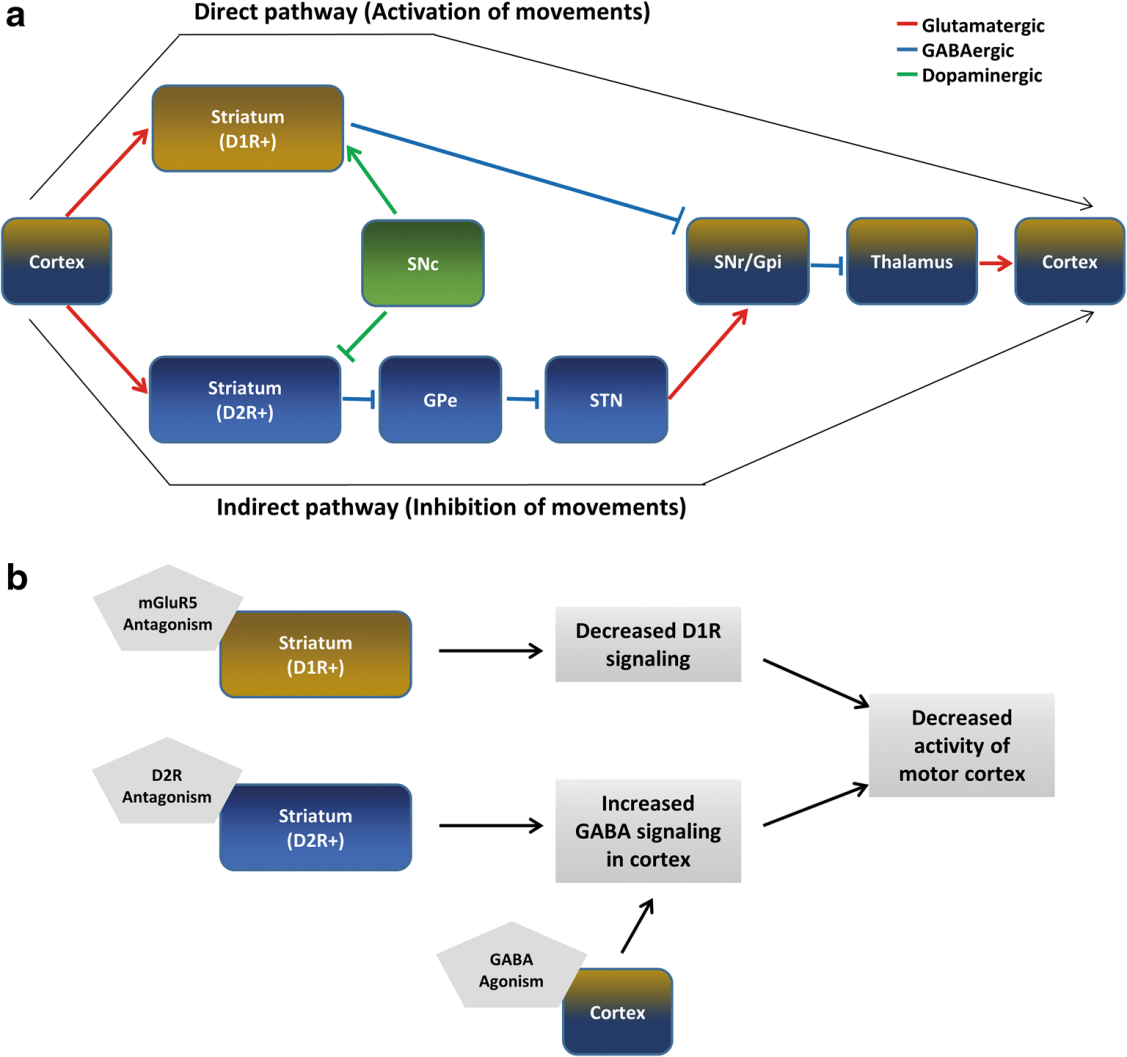
Neural pathways implicated in repetitive behaviors. **a** Schematic drawings of the cortico-basal ganglia-thalamic pathways including the direct and indirect pathways. The direct pathway is represented by *brown color*, while the indirect pathway is represented by *blue color*. The regions that are common in both pathways are represented with *mixed brown* and *blue colors*. Dopaminergic sources from substantia nigra pars compacta (SNc), which activate and inactivate direct and indirect pathways, respectively, are represented by *green color*. The projections from cortex activate both D1R and D2R-expressing GABAergic neurons in the striatum. Then, the D1R-expressing GABAergic neurons in the direct pathway inhibit the internal globus pallidus (GPi) and substantia nigra pars reticulata (SNr). Sequentially, the GABAergic output from GPi and SNr inhibits thalamus and in turn thalamus activates the motor cortex. Thus, the final consequence of the direct pathway is activation of movements. In contrast, D2R-expressing neurons in the indirect pathway inhibit the external globus pallidus (GPe) and GPe inhibits subthalamic nucleus (STN). The STN then activates GPi and SNr, which inhibits thalamus. Hence, the final consequence of indirect pathway is inactivation of movements. **b** Possible neuronal mechanisms of pharmacological rescue of repetitive behaviors. mGluR5 antagonism can inhibit the direct pathway by inhibiting the D1R signaling. GABA agonists can ameliorate elevated E/I balance in the motor cortex of some ASD mouse models, and D2R antagonism may potentiate the GABAergic function in cortical areas

Various brain regions and pathways govern repetitive behaviors; however, the most notable pathway is the cortico-basal ganglia-thalamic pathway (Fig. 1a), which is also involved in the motor activities [42, 43]. For example, human brain imaging studies showed the positive correlation between the volume of basal ganglia compartments such as striatum, and degree of repetitive behavior in ASD patients [44]. In case of animal studies, injecting L-DOPA, a dopamine precursor, and apomorphine, a non-selective dopamine agonist, into the striatum of rats induced stereotypic gnawing behaviors [45]. In addition, a study demonstrated that *Shank3b* ^−/−^ mice, which have abnormal self-grooming behavior, exhibited a reduced cortico-striatal synaptic transmission in medium spiny neurons (MSNs) [34].

The cortico-basal ganglia-thalamic pathway primarily consists of direct D1R-expressing and indirect D2R-expressing pathways (Fig. 1a). D1R-expressing GABAergic neurons in the direct pathway inhibit the internal globus pallidus (GPi) and substantia nigra pars reticulata (SNr). Sequentially, the GABAergic output from GPi to SNr inhibits thalamus and in turn thalamus activates the motor cortex. Thus, the final consequence of direct pathway is activation of movements. In contrast, D2R-expressing neurons inhibit the external globus pallidus (GPe) and GPe inhibits subthalamic nucleus (STN). The STN then activates GPi and SNr, which inhibits thalamus. Hence, the final consequence of indirect pathway is inactivation of movements and possibly affects repetitive behaviors of ASD mouse models. For example, mouse models of 16p11.2 deletion, which occupies relatively high incidence in ASD patients, show decreased grooming, accompanied with increased numbers of D2R-expressing striatal medium spiny neurons (MSNs) and increased spontaneous EPSC in the neurons [46, 47]. Interestingly, these mouse lines also showed increased hanging and circling, suggesting different regulatory mechanisms in indirect pathway among grooming, hanging and circling [47].

It is thought that sub-pathways of the cortico-basal ganglia-thalamic circuit and the dynamic molecular regulation in the sub-pathways are responsible for each class of repetitive behaviors [48–50]. For example, intrastriatal injection of a D1R antagonist, SCH23390, or an NMDA-R antagonist, MK-801, in deer mouse, a well-known jumping mouse, reduced repetitive jumping [51], while injecting apomorphine induced stereotypic grooming and hyperactivity without affecting the jumping [52]. Similarly, chronic administration of the selective serotonergic reuptake inhibitor (SSRI), escitalopram, attenuated the horizontal repetitive movements characteristic of deer mice, without affecting the vertical repetitive movements including jumping, implicating a differential regulation of jumping and other repetitive behaviors [53].

Furthermore, previously uncovered connections related to the cortico-basal ganglia-thalamic circuits are discovered recently. For example, the inhibitory projections, with or without acetylcholine, from external globus pallidus (GPe) to frontal cortex were found [54]. In addition, the same research group demonstrated that a non-canonical function of D2R-expressing neurons in indirect pathway, which activates the motor cortex temporally, suggesting more heterogeneity in the circuits than we expected [55].

An interesting feature of ASD mouse models is that observed abnormal synaptic functions have mostly been found in the hippocampus. In spite of the lack of evidence showing the causal relationship between hippocampus and repetitive behaviors, stimulation of ventral hippocampus with NMDA increased locomotor activity, while its inhibition decreased the locomotor activity [56, 57]. In addition, the hippocampus has connections to several brain regions in cortico-basal ganglia-thalamic circuits, including striatum and amygdala, suggesting the possibility that the hippocampus affects the cortico-basal ganglia-thalamic circuits and possibly repetitive behaviors [58–64].

## Pharmacological rescues of repetitive behaviors

In spite of the limited understanding of the causes of repetitive behaviors, some repetitive behaviors of ASD mouse models were rescued by administering specific treatments. In particular, pharmacological treatments that manipulate the neuronal activity involved in repetitive behaviors are the most salient treatment methods because of their potential to be applied in therapies for human ASD patients.

For this reason, a series of drugs have been tested for ASD mouse models, some of which successively reduced the repetitive behaviors of the mice. Among the many ASD mouse models, the BTBR mice is one of the most frequently studied models and several drugs have reduced their repetitive self-grooming without sedation effects, as we mentioned earlier (Table 1). Their self-grooming was reduced by mGluR5 antagonists or GABA-R agonists [25–27, 65]. Although the involvement of its mechanisms in repetitive behaviors remains unclear, inhibition of mGluR5 expression could activate D1R signaling by stimulating the PKA activity. Accordingly, mGluR5 antagonism possibly inhibits the direct pathway of the cortico-basal ganglia-thalamic circuit [66] (Fig. 1b). In addition, the knockdown of mGluR5 in D1R-expressing cells reduced locomotion activity, supporting the idea [67]. Meanwhile, GABA-R agonists may reduce elevated excitatory/inhibitory (E/I) balance, rescuing overactivation of the motor cortical area, where the final signal of the cortico-basal ganglia-thalamic circuit arrives (Fig. 1b) For example, repetitive behaviors induced by amphetamine was attenuated by GABA-R agonists [68]. In addition, *Fmr1* ^−*/*−^ mice showed hyperexcitability caused by decreased activities of fast-spiking (FS) inhibitory neurons in the somatosensory and barrel cortex, and a GABA-R agonist that reduced the self-grooming of BTBR mice also reduced the locomotor activity and repetitive marble burying of *Fmr1* ^−*/*−^ mice. Moreover, a human fMRI study also reported that the GABA concentration in the supplementary motor area (SMA) was inversely correlated with the excitability of the motor cortex, supporting the relationship between GABAergic function in cortical areas and repetitive behaviors [69].

Another type of drug that reduces repetitive self-grooming is risperidone, which modulates various molecular targets but primarily blocks the D2R, resulting in reduced activity of the indirect pathway. However, reduced activity of the indirect pathway is generally thought to increase repetitive behaviors. Thus, the effect of risperidone, which reduces the repetitive self-grooming of *Cntnap2* ^−*/*−^ mice is somewhat paradoxical. However, it is interesting that systemic injection of haloperidol, a D2R antagonist, also reduced movements and activities of the motor cortex of rats [70]. In addition, D2R overexpression in the striatum enhanced the GABAergic function of the prefrontal cortex (PFC), reducing the excitability of PFC [71]. These results suggest that reduced self-grooming by risperidone may be attributed to the enhancement of GABAergic inhibition of the cortex (Fig. 1b).

## Hub brain regions implicated in both social and repetitive behaviors

**Fig. 2:**
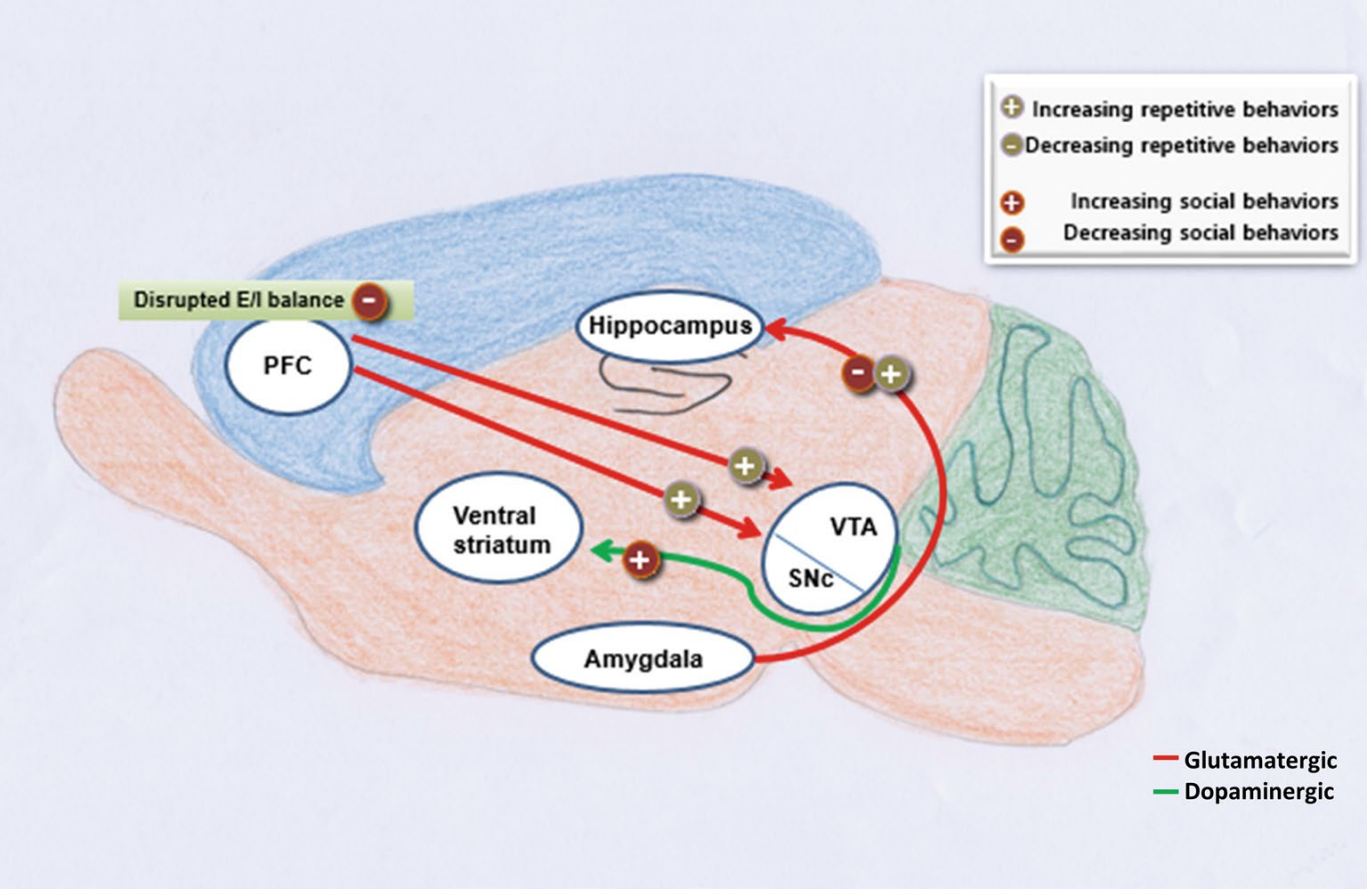
Connections of brain regions implicated in both social and repetitive behaviors. Activation of projection from the amygdala to the hippocampus increases repetitive self-grooming behavior, yet decreasing social behaviors [61]. Optogenetic disruption of E/I balance of the PFC impairs social behaviors [77]. Inhibition of the PFC projection to the VTA reduces repetitive behaviors and activation of the projection from the PFC to the SNc induces hyperactivity [72, 81]. Dopaminergic projections from the VTA to the ventral striatum, a key region implicated in motivation is necessary for social reward [82]. *SNc* substantia nigra pars compacta, *PFC* prefrontal cortex, *VTA* ventral tegmental area

Since the main symptoms of ASD are impairments in social interactions and repetitive behaviors, it may be reasonable to postulate a hub brain region that links the social behaviors and repetitive behaviors. Supporting this view, amygdala may be the most notable candidate region specifically involved in self-grooming. Activation of a population of vesicular glutamate transporter 2 (vGLUT2)-positive glutamatergic neurons in the medial amygdala (MeA) of mice promoted self-grooming while suppressing social interaction. On the other hand, the activation of vesicular GABA transporter (vGAT)-positive GABAergic neurons in the MeA triggered social interaction while suppressing self-grooming [31]. In addition, when the glutamatergic projection from basolateral amygdala (BLA) to the ventral hippocampus of mice was activated, social interaction was reduced while the self-grooming increased [61] (Fig. 2). Thus, it is possible that amygdalo-hippocampal pathway may be highly activated in ASD mouse models.

PFC also can modulate both social and repetitive behaviors. PFC has a projection to the substantia nigra pars compacta (SNc), a dopaminergic source of the striatum. When the projection was activated, it induced hyperactivity and repetitive movement behaviors of mice due to hyperdopaminergia in the striatum [72] (Fig. 2). Moreover, the proper level of dopamine in PFC seems to be required for preventing repetitive behaviors. For example, when an antagonist of dopamine D2 and D3 receptors, sulpiride was injected unilaterally into the PFC of rats pretreated with amphetamine, it induced ipsiversive circling [73]. In another study, after sensitization to cocaine, cocaine injection in rats induced stereotypy with increased PFC dopamine release and decreased the activity of both inhibitory and excitatory projection from medial PFC (mPFC) to SNr [74].

Besides the involvement in repetitive behaviors, PFC also affects social behaviors. For example, lateral prefrontal cortex (LPFC) is important for social information processing and early damage of PFC resulted in impairment of social behaviors [75, 76]. Furthermore, optogenetic activation of mPFC increased E/I balance in that region and impaired social behaviors of the mice [77] (Fig. 2).

Indeed, ASD mouse models often show abnormalities in their PFC*. Scn1a* ^+/−^ mouse, one of autistic mice, showed increased E/I balance in PFC [13]. Another autistic mouse, *Fmr1* ^−*/*−^ mouse, showed hyperconnectivity of layer 5 pyramidal neurons in mPFC [78]. In addition, Neuroligin 2 (NL2) overexpression in PFC of mice increased mIPSC frequency and repetitive jumping, and impaired social behavior of the mice [79]. Thus, impairment in PFC may also cause autistic symptoms in subpopulation of patients.

Ventral tegmental area (VTA) is also responsible for both social and repetitive behaviors. According to a study, after being sensitized to cocaine, VTA glutamate release was increased. This glutamate increase and cocaine-induced stereotypy were rescued by SCH-23390, a D1R antagonist, infusion into VTA [80]. In addition, blocking non-NMDA-R-mediated glutamatergic transmission from PFC to VTA reduced increased self-grooming of rats that was induced by an NMDA-R antagonist, phencyclidine (PCP) [81] (Fig. 2). Since VTA has a dopaminergic projection to PFC, it is likely that the regulation of glutamatergic transmission in PFC by VTA dopaminergic neurons may be important for repetitive self-grooming and motor activities.

As to social behaviors, activity of the dopaminergic projection from VTA to Nucleus Accumbens (NAc), was increased when a mouse confronted with a social objects. Furthermore, optogenetic activation or inhibition of this projection increased or decreased the social interaction time for the subject mice [82]. Thus, abnormal dopaminergic activities in the NAc possibly impair social behaviors and generate repetitive behaviors of ASD mouse models.

## Conclusion

We have discussed the general features of ASD and one of its main symptoms, repetitive behaviors. We then summarized the transgenic or inbred ASD mouse models and their phenotypes focusing on specific repetitive behaviors, electrophysiological properties, and neuronal circuit abnormalities. And then, we summarized pharmacological interventions that used to reduce repetitive behaviors in ASD mouse models. Finally, we discussed hub brain regions possibly involved in both social and repetitive behaviors.

Despite the many studies using ASD mouse models, it is still difficult to identify the brain networks responsible for each repetitive behavior. Moreover, physiological properties of mice with repetitive behaviors are not convergent even in mice with the same repetitive behavior. Although the causal relationships have not yet been proven, organizing the results of the studies provides a glimpse into the relationship between the different brain regions and the corresponding abnormal synaptic properties and repetitive behaviors associated with various ASD mouse models.

Further research should be conducted to investigate the neuronal abnormalities in specific brain regions associated with the repetitive behaviors in ASD mouse models. For that reason, it would be helpful to study the microcircuitry of ASD mouse models in more detail by manipulating specific neuronal populations using pharmacological drugs and conditional KO or optogenetics that currently used in broad brain research area. By doing so, understanding neuronal mechanisms of the repetitive behaviors in ASD would be also helpful to find safer and more effective treatments for each patient with ASD, in a situation that an efficient treatment for ASD symptoms is limiting.

## Abbreviations

BLA: basolateral amygdala
BTBR: BTBR T + tf/J
Cntnap2: contactin-associated protein-like 2
D1R: dopamine D1 receptor
D2R: dopamine D2 receptor
DSM-5: diagnostic and statistical manual of mental disorders
Eif4ebp2: eukaryotic translation initiation factor 4E-binding protein 2
Fmr1: fragile X mental retardation 1
GABA: γ-Aminobutyric acid
Gabrb3: gamma-aminobutyric acid receptor subunit β-3
Grin1: glutamate receptor, ionotropic, N-methyl d-aspartate 1
MeA: medial amygdala
Mecp2: methyl CpG binding protein 2
mGluR5: metabotropic glutamate receptor 5
NMDA-R: N-methyl-d-aspartate glutamate receptor
PSD: postsynaptic density
RBS: repetitive behavior subscales
Scn1a: sodium channel, voltage-gated, type 1α
SERT: serotonin transporter
Shank: SH3 and multiple ankyrin repeat domains
SNpc: substantia nigra pars compacta
vGAT: vesicular GABA transporter
vGLUT2: vesicular glutamate transporter 2
Viaat: vesicular inhibitory amino acid transporter
